# Combined evaluation of ambulatory‐based late potentials and nonsustained ventricular tachycardia to predict arrhythmic events in patients with previous myocardial infarction: A Japanese noninvasive electrocardiographic risk stratification of sudden cardiac death (JANIES) substudy

**DOI:** 10.1111/anec.12803

**Published:** 2020-09-24

**Authors:** Kenichi Hashimoto, Mari Amino, Koichiro Yoshioka, Yuji Kasamaki, Toshio Kinoshita, Takanori Ikeda

**Affiliations:** ^1^ Department of General Medicine National Defense Medical College Tokorozawa Japan; ^2^ Department of Cardiovascular Medicine Tokai University Isehara Japan; ^3^ Department of General Medicine Kanazawa Medical University Himi Municipal Hospital Himi Japan; ^4^ Department of Cardiovascular Medicine Faculty of Medicine Toho University Tokyo Japan

**Keywords:** arrhythmic risk stratification, Holter electrocardiography, late potential, nonsustained ventricular tachycardia, sudden cardiac death, ventricular arrhythmia

## Abstract

**Background:**

Noninvasive electrocardiographic (ECG) markers are promising arrhythmic risk stratification tools for identifying sudden cardiac death. However, little is known about the usefulness of noninvasive ECG markers derived from ambulatory ECGs (AECG) in patients with previous myocardial infarction (pMI). We aimed to determine whether the ECG markers derived from AECG can predict serious cardiac events in patients with pMI.

**Methods:**

We prospectively analyzed 104 patients with pMI (88 males, age 66 ± 11 years), evaluating late potentials (LPs), heart rate turbulence, and nonsustained ventricular tachycardia (NSVT) derived from AECG. The primary endpoint was the documentation of ventricular fibrillation or sustained ventricular tachycardia.

**Results:**

Eleven patients reached the primary endpoint during a follow‐up period of 25 ± 9.5 months. Of the 104 patients enrolled in this study, LP positive in worst values (w‐LPs) and NSVT were observed in 25 patients, respectively. In the arrhythmic event group, the worst LP values and/or NSVT were found in eight patients (7.6%). The positive predictive and negative predictive values of the combined assessment with w‐LPs and NSVT were 56% and 94%, respectively, for predicting ventricular lethal arrhythmia. Kaplan–Meier analysis demonstrated that the combination of w‐LPs and NSVT had a poorer event‐free period than negative LPs (*p* < .0001). In the multivariate analysis, the combined assessment of w‐LPs and NSVT was a significant predictor of arrhythmic events (hazard ratio = 14.1, 95% confidence intervals: 3.4–58.9, *p* < .0001).

**Conclusion:**

Combined evaluation of w‐LPs and NSVT was a powerful risk stratification strategy for predicting arrhythmia that can lead to sudden cardiac death in patients with pMI.

## 1 INTRODUCTION

There are 230,000–350,000 cases of sudden cardiac death (SCD) annually in the United States (Al‐Khatib et al., 2018), with coronary artery disease (CAD) being a major cause having rates between 65% and 80% (Huikuri, Castellanos, & Myerburg, 2001; Junttila et al., 2016). Insertion of an implantable cardioverter–defibrillator (ICD) is common for preventing arrhythmic SCD. Although the ICD implantation rate has increased over the last two decades, SCD has not reduced (Benjamin et al., 2017). Recent reports indicated that the left ventricular ejection fraction (LVEF), nonsustained ventricular tachycardia (NSVT), and programmed stimulation on electrophysiology studies provide a high level of evidence for identifying arrhythmic SCD risk, necessitating ICD implantation (Al‐Khatib et al., 2018; Priori et al., 2015; Scirica et al., 2010). However, these markers do not cover patients at risk for aborted SCD.

Ventricular late potentials (LPs) on a signal‐averaged electrocardiogram (SAECG) are useful for identifying the risk of arrhythmia leading to SCD in patients with previous myocardial infarction (pMI) who have reduced cardiac function (Gomes et al., 1987; Malik et al., 1992; Steinberg, Regan, Sciacca, Bigger, & Fleiss, 1992). Since the 2000s, primary percutaneous coronary intervention (PCI) has been performed during the earlier stages of cardiac dysfunction and drug‐eluting stents have been considered the gold standard for PCI. Therefore, more patients with pMI who performed PCI after 2000s have a preserved LVEF rather than 1980–1990s. However, most investigations regarding risk stratification for fatal arrhythmic events using SAECG were done before 2000s. Recently, LPs have been measured for 24 hr using high‐resolution ambulatory electrocardiogram (AECG) systems (Amino et al., 2019; Hashimoto et al., 2018). The use of ambulatory‐based LPs for identifying lethal arrhythmias in patients with pMI is not fully understood.

Noninvasive electrocardiographic (ECG) markers, such as heart rate turbulence (HRT) and NSVT, are useful for predicting cardiac events in various clinical settings. However, the predictive value of LPs, HRT, and NSVT alone is limited, especially if the positive predictive value (PPV) is low (Ikeda et al., 2000; Miwa et al., 2012). We hypothesized that a combined assessment of these indices, including ambulatory‐based LPs, could predict lethal arrhythmias. Therefore, this study aimed to prospectively evaluate the usefulness of ambulatory‐based LP measurements and determine whether simultaneous assessment of noninvasive ECG risk stratification factors, such as ambulatory‐based LPs, HRT, and NSVT, can be used to predict fatal arrhythmic events, such as sustained ventricular tachycardia (VT) or ventricular fibrillation (Vf) in patients with pMI.

## 2 METHODS

### 2.1 Study design and ethics

This study was a substudy of the Japanese Noninvasive Electrocardiographic Risk Stratification for Prediction of Sudden Cardiac Death (JANIES) study (Kinoshita et al., 2020). The JANIES study was designed as a multicenter, observational, prospective cohort study and evaluated noninvasive ECG indices, such as LPs, HRT, and NSVT, which can be analyzed simultaneously using a 24‐hr digital AECG, to predict cardiac mortality and/or lethal arrhythmias in high‐risk patients. Participants in the substudy were enrolled between April 2012 and March 2015. Inclusion and exclusion criteria used in this study are reported elsewhere (Kinoshita et al., 2020). Briefly, the patients with structural or idiopathic cardiac disorder who underwent 24‐hr high‐resolution digital ambulatory monitoring were included. Exclusion criteria were (a) patients with cardiomyopathy, (b) arrhythmogenic right ventricular cardiomyopathy, (c) persistent atrial fibrillation or flutter, (d) right or left bundle branch block and intraventricular conduction delay, (e) permanent pacing, (f) atrio‐ventricular block II–III degree, and (g) patients in whom all ECG markers could not be simultaneously measured. For this study, the exclusion criteria also involved the presence of structural heart disease except for pMI and idiopathic cardiac disorder. In this substudy, 137 patients with pMI who underwent AECG using a system manufactured by Spider View (Ela, Paris, France) were included. Eventually, 33 patients were excluded from our study since the noninvasive ECG data could not be analyzed due to noise, missing follow‐up data, or unknown cause; thus, 104 patients (mean age: 66.7 ± 11.7 years old, 88 men) with a history of MI in the previous 1 month to 8 years (median, 42; interquartile range, [1, 101] months) were analyzed. Patient demographics are presented in Table 1. The mean LVEF of the study cohort was 58.5 [50.0, 65.9]. Therefore, most patients enrolled in this study had a preserved cardiac systolic function. The study was conducted in accordance with the Declaration of Helsinki and was approved by the Competent Authorities and Ethics Committees of the participating centers. All patients provided informed consent. The JANIES study was approved by the Ethics Committee of Toho University Omori Medical Center (approved number 23‐135) and registered with the UMIN Clinical Trials Registry (UMIN000007683).

**Table 1:** Baseline characteristics of the study patients (*n* = 104)

Demographics	Arrhythmic event group (*n* = 11)	Nonarrhythmic event group (*n* = 93)	*p*
Age (year)	64.5 ± 10.4	66.9 ± 11.9	.33
Sex: male, *n* (%)	10 (90.9)	77 (83)	.21
Hypertension, *n* (%)	9 (83)	77 (83)	.94
Dyslipidemia, *n* (%)	5 (46)	64 (69)	.12
Diabetes mellitus, *n* (%)	6 (55)	39 (42)	.43
PCI	9 (82)	78 (84)	.86
CABG	2 (18)	15 (16)	.94
Coronary culprit lesion			
RCA	2 (18)	38 (41)	.5
LAD	8 (73)	45 (48)	.14
Cx	1 (9)	10 (11)	.67
Echocardiographic data			
LVEF (%)	48.0 ± 4.8	57.9 ± 11.6	.045
LVDD (mm)	59.3 ± 14.1	50.0 ± 7.3	.029
12‐lead electrocardiogram data			
QRS duration	98.6 ± 12.8	103.8 ± 12.1	.095
QTc interval	435.1 ± 22.8	445.6 ± 19.4	.06
NYHA functional class			
NYHA I (%)	7 (64)	76 (82)	.27
NYHA II (%)	3 (27)	15 (16)	.4
NYHA III (%)	1 (9.1)	2 (2.2)	.29
NYHA IV (%)	0 (0)	0 (0)	—
Therapy			
β‐blocker (%)	9 (82)	69 (74)	.73
RAS inhibitor (%)	4 (64)	58 (63)	.09
CCB (%)	30 (27)	27 (29)	.61
Diuretic (%)	4 (36)	28 (30)	.67
Amiodarone (%)	4 (36)	7 (7.5)	.003
IB (%)	0 (0)	3 (6.5)	.51
IIC (%)	0 (0)	1 (1.1)	.89
ICD implantation	6 (54)	3 (3)	<.001
Holter ECG findings			
b‐LPs	4 (36)	13 (14)	.079
w‐LPs	7 (63)	18 (15)	.002
Abnormal HRT	5 (45)	27 (29)	.26
NSVT document	6 (55)	19 (20)	.022

Note

Data given as *n* (%) or means.

Abbreviations: b‐LPs, LP positive in best value; CABG, coronary artery angioplasty; CCB, calcium channel blockers; Cx, left circumflex artery; LAD, left anterior descending artery; LP, late potential; LVDD, left ventricular dimension diameter; LVEF, left ventricular ejection fraction; NYHA, New York Heart Association; PCI, percutaneous coronary angioplasty; RAS, renin–angiotensin system; RCA, right coronary artery; w‐LPs, LP positive in worst value.

### 2.2 AECG recordings

Data derived from the 24‐hr digital AECG system were analyzed to detect NSVT and specific noninvasive ECG markers, such as the 24‐hr SAECG (24‐hr SAECG) and HRT. All patients underwent 24‐hr digital AECG with ordinary daily activities. The presence of NSVT was defined as ≥3 consecutive ventricular premature contractions (VPC) at >100 beats/min, as previously reported (de Sousa, Morillo, Rabelo, Nogueira Filho, & Ribeiro, 2008). All patients underwent 24‐hr AECG recording after three weeks from the onset of MI to avoid acute‐phase electrical instability.

### 2.3 Measurement of ambulatory‐based LPs

Late potentials were recorded for all patients with PMI using the AECG system (Spider View; Ela, Paris, France). ECG data were obtained at a sampling rate of 1,000 Hz and 16‐bit A/D conversion. For LPs measurement, ECG data were filtered and ranged from 40 to 250 Hz. Orthogonal X, Y, and Z bipolar leads with silver–silver chloride electrodes (Blue SENSOR^®^; METS) were used for all LP recordings. LP parameters were automatically measured 6 times (0:00–4:00; 4:00–8:00; 8:00–12:00; 12:00–16:00; 16:00–20:00; and 20:00–24:00) over a 24‐hr time period; parameters were manually edited by the cardiologist using Syne Scope (SORIN GROUP). The cardiologists were blinded to patient outcomes. Three parameters of the 24h‐LPs were evaluated, including the filtered QRS duration (fQRS), the duration of low‐amplitude signals < 40 μV in the terminal filtered QRS complex (LAS_40_), and root‐mean‐square voltage of the terminal 40 ms in the fQRS complex (RMS_40_) in patients with pMI. LPs were considered positive when any two of the following three criteria were met: fQRS > 114 ms, RMS_40_ < 20 μV, and LAS_40_ > 38 ms (Breithardt et al., 1991). LP positive in best values (b‐LPs) were defined as more than two of the three LP parameters being positive when the RMS_40_ was largest during each period. While LP positive in worst values (w‐LPs) were defined as more than two of the three LP parameters being positive when the RMS_40_ was smallest during each period.

### 2.4 HRT measurements

Heart rate turbulence was measured from the AECG, according to a previously established protocol (Bauer et al., 2008). It was measured if more than one determinate VPC occurred. HRT was evaluated by turbulence onset (TO) and turbulence slope (TS). TO reflects the early‐phase sinus rhythm acceleration after VPC and is followed by TS, which represents the compensatory deceleration phase after VPC. TS was calculated as the steepest regression over any five consecutive sinus rhythm RR intervals after VPC and within 15 sinus rhythms (Bauer et al., 2008). TO ≥ 0% and TS ≤ 2.5 ms/RR intervals were considered abnormal. HRT was considered positive when the TO and TS were abnormal; it was considered negative when TO and/or TS were abnormal or when HRT could not be calculated due to a lacking VPC.

### 2.5 Assessment of other clinical parameters

We prospectively evaluated age, sex, hypertension, dyslipidemia, diabetes mellitus history, and EF using the modified Simpson method (Lang et al., 2015), and left ventricular dimension diameter (LVDD) using echocardiography. A reduced EF was defined as an EF < 40%. Also, QRS duration and QTc intervals were measured from 12‐lead ECG at the period near when the 24‐hr AECG recordings were performed.

The QTc intervals were calculated using Bazett formula (Bazett, 1920). The use of medications, such as β‐blockers, renin–angiotensin system inhibitors (including angiotensin‐converting enzyme inhibitors/angiotensin receptor blockers), calcium blockers, diuretics, and class III antiarrhythmic drugs (e.g., amiodarone) or class Ib and IIc antiarrhythmic drugs, was evaluated.

### 2.6 Follow‐up and study endpoints

The primary endpoint was the occurrence of fatal arrhythmic events, such as Vf or sustained VT. The causes of death were confirmed from medical or autopsy records, and from the primary doctors or those who had witnessed the death. For fatal arrhythmic events, the records were verified from ECG monitoring detected in the hospital room, 24‐hr Holter electrocardiograms recorded from the hospital, or by confirming the use of an ICD. In patients with ICDs, an appropriate shock delivery for VF events and antitachycardia pacing for sustained VT were included in the fatal arrhythmic events. Follow‐up data were collected at 6‐month intervals until September 2015.

### 2.7 Statistical analyses

Data are presented as mean ± standard deviation for normally distributed continuous variables and as medians [interquartile range: 25th–75th percentile] for non‐normally distributed variables. Patient characteristics were compared using the chi‐square test for categorical variables and Student's *t* test for continuous and parametric data and by the Wilcoxon rank‐sum test for nonparametric data. Comparisons of the b‐LPs and w‐LPs parameters over 24 hr were conducted using Student's *t* test or Wilcoxon signed‐rank test, as appropriate. Friedman's analysis of variance on rank was used for the comparisons of fQRS for each time interval. Cox univariate and multivariate regression analyses were performed to analyze associations between the endpoints and clinical parameters. Cardiac event‐free rates in each category associated with noninvasive risk stratification markers were calculated using Kaplan–Meier methods; differences in cardiac event‐free rates were calculated using the log‐rank test. All statistical analyses were performed using SPSS version 25 (IBM Corp). *t* tests were 2‐sided, and P values < 0.05 were considered statistically significant.

## 3 RESULTS

### 3.1 Baseline characteristics

During the mean follow‐up period of 25 ± 9.5 months, 11 patients reached the primary endpoint. Baseline characteristics of this study population are described in Table 1. In echocardiographic data, LVEF was lower in arrhythmic event group; conversely, LVDD was larger in nonarrhythmic event group (Table 1).

### 3.2 LP parameters fluctuation throughout 24 hr

**Figure 1:**
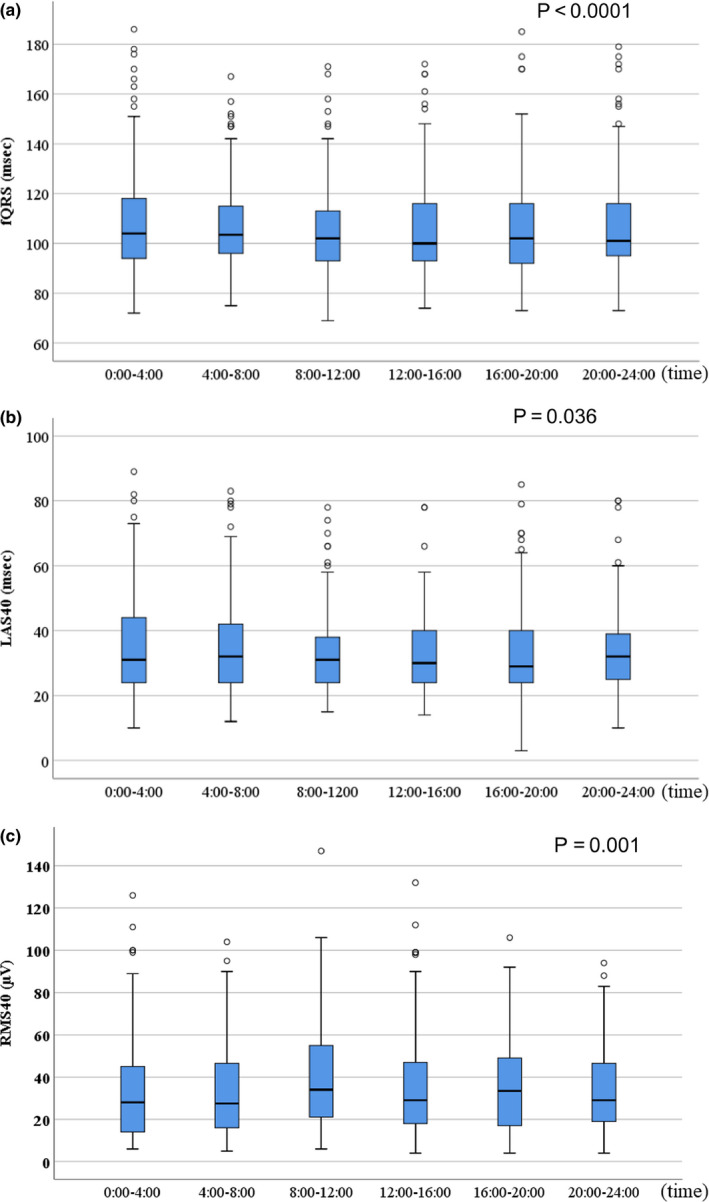
Late potential parameters significantly fluctuated over a 24‐hr time period. (a) The median fQRS was the longest between 0:00 and 4:00; the length of the fQRS gradually decreased after this time point. The shortest fQRS was observed between 12:00 and 16:00 (*p* < .0001). (b) The median LAS_40_ was the longest between 4:00 and 8:00. The shortest LAS_40_ was observed between 12:00 and 16:00 (*p* = .036). (c) The median LAS_40_ was the shortest between 8:00 and 12:00. The longest LAS_40_ was observed between 0:00 and 4:00 (*p* = .001). fQRS, filtered QRS; LAS_40_, low‐amplitude signals < 40 μV in the terminal filtered QRS complex; RMS_40_, root‐mean‐square voltage of the terminal 40 ms in the filtered QRS complex

All LP parameters (fQRS, LAS_40_, and RMS_40_) significantly fluctuated over the 24‐hr period. The median fQRS was longest between 0:00 and 4:00; conversely, it was shortest between 12:00 and 16:00 (*p* < .0001) (Figure 1). Additionally, the median LAS_40_ was longest between 4:00 and 8:00; conversely, it was shortest between 12:00 and 16:00 (*p* < .036). Conversely, the median RMS_40_ was the largest between 8:00 and 12:00 and the smallest between 0:00 and 4:00 (*p* = .001) (Figure 1). The rate of w‐LPs in the arrhythmic event group was significantly higher than the b‐LPs positive rate in the nonarrhythmic event group (63% vs. 15%, respectively; *p* = .002). However, there was no difference between groups regarding b‐LPs‐positive rate (36% vs. 14%, in the arrhythmic and nonarrhythmic group, respectively; *p* = .079) (Table 1). The worst value of fQRS and LAS_40_ was significantly higher than the best value of fQRS and LAS_40_ (*p* < .001, in both groups). The worst value of RMS_40_ was significantly smaller than the best value of RMS_40_ (*p* < .001). The noise level of best value of LP was significantly higher than that of the worst value of LP. (Table 2).

**Table 2:** The best and worst values of LP parameters

	The best value of LP	The worst value of LP	*p*
fQRS (ms)	96.0 [89.0, 111.5]	109.5 [99.5, 124.0]	<.001
LMS_40_ (ms)	28.0 [22.0, 36.5]	34.5 [27.0, 49.5]	<.001
RAS_40_ (μV)	42.5 [23.0, 71.0]	25.5 [11.6, 36.0]	<.001
Noise (μV)	0.59 [0.3, 0.73]	0.34 [0.26, 0.49]	<.001

Note

Data given as median [interquartile range].

Abbreviations: fQRS, filtered QRS duration; LAS40, duration of low‐amplitude signals < 40 μV in the terminal filtered QRS complex; LP, late potentials; RMS40, root‐mean‐square voltage of the terminal 40 ms in the filtered QRS complex.

### 3.3 Association between ECG risk stratification markers and end arrhythmic events

**Figure 2:**
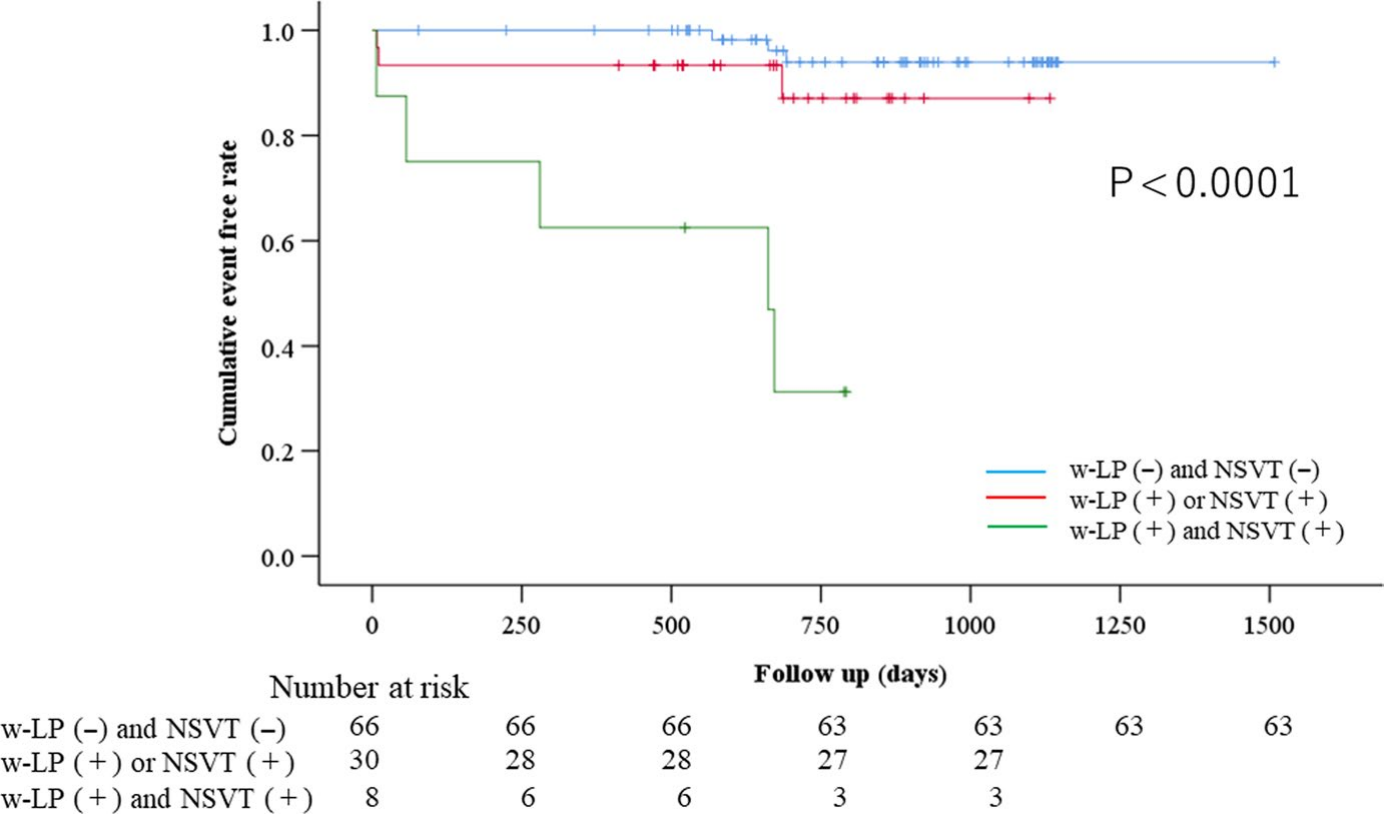
Kaplan–Meier curves for serious cardiac events based on the combination assessment of w‐LPs and NSVT. The combination of w‐LPs‐positive values and NSVT‐positive values was associated with a poorer event‐free rate (*p* < .0001). HRT, heart rate turbulence; NSVT, nonsustained ventricular tachycardia; w‐LPs, the worst value of late potential

The clinical characteristics of the patients are shown in Table 3. Vf was observed in three patients, and sustained VT was documented in eight patients. Ten of the eleven patients had a NYHA cardiac functional classification of I or II. EF < 40% was documented in four patients (Nos 1, 2, 9, and 11). Five patients died due to lethal arrhythmia in sustained VT and Vf (Nos 2, 3, and 11), or heart failure (Nos 4 and 5). Only three patients (Nos 2, 5, and 7) developed arrhythmic events without w‐LPs positive and/or documented NSVT. Table 4 demonstrates the predictive values and univariate Cox regression analysis and shows the association between the single and combined assessments and risk ECG markers (b‐LPs, w‐LPs, EF, HRT, and NSVT). The w‐LPs, EF, and NSVT were significant indices to predict lethal arrhythmia, but HRT was not significant. When the w‐LPs and NSVT values were combined, the highest PPV (56%), highest positive likelihood ratios (PLR) (10.6), and highest predictive accuracy (PA) (90%) were obtained; the hazard ratio (HR) was 13.5 ([3.6–51.7], *p* < .0001). Despite adjusting for age and EF, the combined assessment (with w‐LPs and NSVT) was a significant predictor of arrhythmic events (HR = 14.1, 95% confidence interval [CI]: 3.4–58.9, *p* < .0001) (Table 5). Also, w‐LPs + NSVT was a significant factor among noninvasive ECG markers by multivariate Cox regression analysis (HR = 17.6 [1.4–245.8] (Table S1). Moreover, comparison among combined noninvasive markers including EF was performed. As a result, w‐LPs + NSVT was also an independent factor (HR: 12.2 [3.0–49.8], *p* < .0001) (Table S2). Kaplan–Meier analysis demonstrated that both w‐LPs and NSVT positivity were associated with a significantly lower event‐free rate than other combinations of w‐LPs and HRT results (*p* < .0001) (Figure 2). Moreover, we performed the analysis by Kaplan–Meier method between reduced or with preserved EF among three categories (w‐LPs(−) and NSVT (−); w‐LPs(+) or NSVT (+); w‐LPs(+) and NSVT (+)). In all these categories, there were no significant differences between reduced (EF < 50%) or preserved EF (EF ≤ 50%) for cumulative event‐free rate by log‐rank test (Figures S1–S3).

**Table 3:** Characteristics of patients with previous myocardial infarction that developed an arrhythmic event

Pt no	Age/gender	NYHA	EF	LVDD	NIEP	ICD	Time to event (days) from AECG recordings	Arrhythmic event	Outcome
1	71/M	1	30.9	61.9	HRT, NSVT	Yes	11	SVT	
2	63/F	1	31.5	82.9	HRT	No	692	SVT	Arrhythmic death
3	71/M	1	53.4	54.0	w‐LPs	No	685	Vf	Arrhythmic death
4	58/M	3	49.0	63.8	b‐LPs, w‐LPs, HRT, NSVT	No	672	SVT	Death due to heart failure
5	47M	2	44.5	61.5	HRT	No	662	Vf	Death due to heart failure
6	86/M	2	66.5	43.8	b‐LPs, w‐LPs, NSVT	Yes	57	SVT	
7	59/M	1	60.5	42.8	None	No	568	SVT	
8	70/M	1	57.0	60.0	b‐LPs, w‐LPs	No	8	SVT	
9	68/M	2	23.7	79.0	b‐LPs, w‐LPs, NSVT	No	7	SVT	
10	62/M	1	74.3	38.0	w‐LPs, HRT, NSVT	Yes	280	SVT	
11	54/M	1	37.4	64.8	w‐LPs, HRT, NSVT	No	662	Vf	Arrhythmic death

Abbreviations: AECG, ambulatory electrocardiogram; b‐LP, LP positive in best value; EF, ejection fraction; F, female; HRT, heart rate turbulence; ICD, implantable cardioverter– defibrillator; M, male; NSVT, nonsustained ventricular tachycardia; NYHA, New York Heart Association; SVT, sustained ventricular tachycardia; Vf, ventricular fibrillation; w‐LPs, LP positive in worst value.

**Table 4:** Predictive values and univariate Cox regression analysis associated with ambulatory electrocardiographic parameters used alone and in combination with arrhythmic event

	Sensitivity (%)	Specificity (%)	PPV (%)	NPV (%)	P‐LRs	N‐LRs	PA (%)	RH (95% CI)	*p*
b‐LPs	27	86	20	91	1.95	0.84	79	1.9 (0.4–9.4)	.41
w‐LPs	63	80	28	95	3.28	0.45	79	8.2 (2.0–33.5)	.003
LVEF	36	91	33	92	4.23	0.7	86	4.9 (1.4–16.7)	.018
HRT	55	70	18	93	1.81	0.65	68	2.4 (0.78–7.50)	.12
NSVT	54	82	26	94	2.98	0.55	79	3.60 (1.6–11.2)	.027
w‐LPs＋LVEF	18	98	50	92	9.2	0.83	90	6.6 (1.4–30.2)	.016
LVEF＋NSVT	27	96	50	92	8.45	0.75	89	9.52 (2.5–35.9)	.001
w‐LPs＋NSVT	45	96	56	94	10.6	0.56	90	13.5 (3.6–51.7)	<.0001

Abbreviations: HRT, heart rate turbulence; LP, late potential; LVEF, left ventricular ejection fraction; N‐LRs, negative likelihood ratios; NPV, negative predictive value; NSVT, nonsustained ventricular tachycardia; PA, predictive accuracy; P‐LRs, positive likelihood ratios; PPV, positive predictive value; RH, relative hazard; w‐LPs, LP positive in worst value.

**Table 5:** Result of multivariate Cox regression analysis

	Multivariate analysis	
	Hazard ratio (95% CI)	*p*
Age	1.02 (0.96–1.1)	.44
LVEF	6.2 (1.6–24.4)	.01
w‐LPs ＋ NSVT	14.1 (3.4–58.9)	<.0001

Abbreviations: HRT, heart rate turbulence; LP, late potential; LVEF, left ventricular ejection fraction; NSVT, nonsustained ventricular tachycardia; w‐LPs, LP positive in worst value.

## 4 DISCUSSION

There were significant daily fluctuations in LP parameters in patients with pMI. Risk assessment, with consideration to diurnal variations of LP, was useful for predicting serious cardiac events. The combined evaluation of w‐LPs and NSVT was a powerful risk stratification strategy for predicting fatal arrhythmic events after adjusting for age and EF. The results of this study included up‐to‐date information available on noninvasive risk stratification strategy in the contemporary ela, because PCI using drug‐eluting stent tended to be performed after 2000s. As such, most patients included in this study had preserved systolic function.

### 4.1 24h‐LP as a predictor of arrhythmic events

In this study, LP parameters significantly worsened during the night and improved during the daytime (Table 2 and Figure 1). The sensitivity, PPV, negative predictive value (NPV), and PLR among the w‐LPs were higher than those among the b‐LPs. Therefore, 24h‐LP can be a useful risk stratification marker for identifying arrhythmic events, compared to b‐LPs. Amino et al. reported that LPs in patients with pMI fluctuated daily (Amino et al., 2019) and that w‐LP values could independently predict cardiac events, resulting in rehospitalization (odds ratio = 2.26). Their results are similar to our findings. We also demonstrated that the assessment of w‐LPs was useful for predicting arrhythmic events in patients with ischemic heart disease (IHD). A new point in our study was the endpoint settings of fatal arrhythmia. In structural heart disease such as IHD, it has been reported that sympathetic activity, HR acceleration, transient ischemia, and body position can affect LP values (Chamiec, Kułakowski, & Ceremuzyński, 1995; Goldberger, Ahmed, Parker, & Kadish, 1994; Kremers, Black, Lange, Wells, & Solo, 1990; Yoshioka et al., 2015). Conversely, Abe et al. (2012) reported that, in patients with Brugada syndrome, LP parameters demonstrated circadian variations, which strongly correlated with parasympathetic activity. Patients with Brugada syndrome are speculated to have different pathologies of LP than those with structural heart diseases, including IHD. The precise mechanisms of daily fluctuation in LP parameters among patients with IHD are still unknown. Further research is necessary to evaluate the precise mechanisms underlying the daily fluctuations in LP parameters.

### 4.2 NSVT as a predictor of arrhythmic events

Nonsustained ventricular tachycardia can potentially cause lethal arrhythmia. Lethal arrhythmias, such as VT and/or Vf, finally occurred via VPC (based on electrophysiological substrate) and modulators such as autonomic nerve activity, electrolyte imbalance, and age (Ikeda, Yusu, Nakamura, & Yoshino, 2007). Several studies have demonstrated that NSVT is useful for predicting lethal arrhythmia in patients with pMI with reduced EF (Farrell et al., 1991; Maggioni et al., 1993). Recent AHA (Al‐Khatib et al., 2018) and ESC (Priori et al., 2015) guidelines indicated that NSVT derived from AECG should be considered for ICD implantation. Recently, it was reported that the risk PPV of NSVT for arrhythmic events in reduced EF patients was low (range: 4%–16%); the NPV, on the other hand, was high (82%–97%). In this study, the NSVT was a statistically significant index for predicting arrhythmic events (HR: 3.6, 95% CI: 1.6–11.2, *p* = .027). Furthermore, the NPV was extremely high (94%), and the PPV was very low (26%) (Table 4). Therefore, in patients with pMI, NSVT alone is limited in its ability to predict arrhythmic events.

### 4.3 HRT as a predictor of arrhythmic events

In our study, HRT was not a useful risk stratification marker. It has been reported that HRT was evidenced by several papers for identifying cardiac death or arrhythmic events in patients with pMI (Disertori, Masè, Rigoni, Nollo, & Ravelli, 2016). The exact reason why HRT was not a significant factor in this study is difficult to certify. It is speculated that this is due to the patient population in this study which had preserved systolic function. In most of the previous studies of HRT in patients with MI population, the subjects had low EF (30%–49%), except ISAR risk study (EF 56%) (Bauer et al., 2009). Most recently, Gatzoulis et al reported a novel arrhythmic risk stratification using noninvasive ECG risk factors in patients with preserved EF post‐MI (median EF, 50.8, interquartile range, [45, 55]). In their algorithm, if at least one index (LPs, numbers of VPCs, NSVT, QTc prolongation, HRT, deceleration capacity, or heart rate variability) was positive, then they performed electrophysiological test for provoking ventricular arrhythmia. In case of inducible VT, then ICD was implanted to prevent SCD. This algorithm yielded excellent sensitivity, specificity, and NPV. In subanalysis of this study, LPs and NSVT‐positive rate were significantly higher in the with inducible VT group than in the without inducible VT group. However, HRT was not significant between with inducible VT and without inducible VT. Moreover, none of patients was HRT‐positive among nine patients who received appropriate ICD shock delivery due to spontaneous lethal arrhythmia. The results of their investigation partially support our data. Indeed, in our study data, HRT was positive in 6 patients out of 11 patients who developed VT or Vf (Table 3). Five of these six patients with arrhythmic events had low EF under 50% (Nos 1, 2, 4, 5, and 11). On the other hand, Bonnemeier et al reported that early reperfusion by PCI from TIMI 2 to TIMI 3 restored HRT parameter (Bonnemeier et al., 2003). Also, they reported that there was positive correlation between EF and TS; then, TS could be restored according to EF improvement. In our study, all of the patients received PCI or coronary artery bypass angioplasty (Table 1) in the early phase of MI. This situation can be influenced by the fact that HRT was not an independent marker for patients with MI who have relatively preserved EF.

### 4.4 Combined evaluation of noninvasive risk stratification markers

The combined evaluation of w‐LPs and NSVT was a powerful risk stratification strategy for predicting fatal arrhythmic events after adjusting for the age and EF (Table 5). To the best of our knowledge, this is the first study to evaluate the usefulness of combined assessment with LP measured for 24 hr and NSVT for identifying fatal arrhythmias. It was previously reported that a combined assessment of LPs recorded in real time and NSVT was useful for predicting serious arrhythmic events (el‐Sherif et al., 1995; Kuchar, Thorburn, & Sammel, 1987). The PPV of these combined assessments was between 24% and 31% (el‐Sherif et al., 1995; Kuchar et al., 1987). Real‐time LP recordings are usually performed during the daytime and last approximately 15 min. We also demonstrated the usefulness of assessing the fluctuations in LP parameters for arrhythmic risk stratification in patients with pMI. In our study, the PPV of the combined evaluation of w‐LPs and NSVT (56%) was superior to the PPVs reported in previous studies (el‐Sherif et al., 1995; Kuchar et al., 1987). On the other hand, our study indicated that risk markers, such as w‐LPs, EF, HRT, and NSVT, had limited applicability in clinical settings; this was because the PPVs were very low (18%–33%) and the NPVs were very high (91%–95%); this is consistent with previous studies (Table 4) (eL‐Sherif et al., 1995; Ikeda et al., 2000; Miwa et al., 2012). In this study, we demonstrated that the PPV of the combination of w‐LPs and NSVT was higher than other single‐use or combination ECG indices. Furthermore, the PLRs and PA were also higher than other single‐use or combination ECG indices. There were no significant differences between reduced (EF < 50%) or preserved LVEF (EF ≥ 50%) for cumulative event‐free rate by Kaplan–Meier method (Figures S1–S3). Therefore, there is a possibility that the combination of w‐LP and NSVT is useful for risk stratification tool regardless of the EF. Also, multivariate Cox regression analysis demonstrated combination of w‐LPs and NSVT had a higher hazard ratio than other single‐use or combination indices (Tables S1 and S2). ICD insertion should be considered to prevent SCD when the w‐LPs and NSVT are positive in patients with pMI.

### 4.5 Limitations

This study has a few limitations. First, the sample size was small, which limited the power of the study. The JANIES study used three commercially available AECG systems (Spider View; SCM 8000; Fukuda Denshi.; Mars, GE Healthcare Inc.). In this substudy, the enrolled patients were examined using the Spider View only. Because the algorithm for the LPs filters characteristic differences depended on the device, we did not combine the analyses for the LP parameters.

Second, we did not include the analyses of repolarization abnormality indices, such as the T‐wave variability (TWV). It has been reported that TWV is a useful marker for identifying the risk of lethal arrhythmia, which can lead to SCD in patients with IHD (Couderc, Zareba, McNitt, Maison‐Blanche, & Moss, 2007). Future prospective studies should include a larger sample size and analysis of the repolarization abnormality index.

Third, our study results demonstrate that the noise level of b‐LPs was significantly higher than that of w‐LPs. There is a possibility that the noise influenced the value of LPs parameters. Maounis et al. had investigated the SAECG parameter changes in each noise level as low (under 0.3 μV), intermediate (0.31–0.59 μV), and high (0.6–0.74 μV) in 83 patients with heart diseases including 40 with IHD. As a result, the SAECG parameters were significantly getting better with higher noise levels. Therefore, they mentioned that the noise level determines the QRS offset. When the noise level increased, the offset of QRS is shifted earlier in timing, compared to lower noise levels. Then fQRS could be shortened. Similarly, when the noise level was higher, the LAS_40_ was shortened and RAS_40_ was increased. In this study, LPs parameters showed daily fluctuation. In patients with IHD, it remains unknown whether SAECG parameters have diurnal variations or only daily fluctuations due to confounders such as a noise. In future studies, it is necessary to perform multivariate analysis for seeking the strongest factor which influences SAECG parameters including the noise level, body position HR, and autonomic nervous activity.

In conclusion, the combined evaluation of w‐LPs and HRT derived from digital ambulatory‐based ECG systems can be a powerful risk stratification strategy for predicting fatal arrhythmias, such as VT or Vf in patients with pMI with relatively preserved EF.

## CONFLICT OF INTEREST

All authors have no conflicts of interest in connection with this article.

## ETHICAL APPROVAL

The study was conducted in accordance with the Declaration of Helsinki and was approved by the Competent Authorities and Ethics Committees of the participating centers. All patients provided informed consent. The JANIES study was approved by the Ethics Committee of Toho University Omori Medical Center (approved number 23‐135) and registered with the UMIN Clinical Trials Registry (UMIN000007683).

## Author contributions

All authors reviewed and approved the manuscript. Directed this study: T. Ikeda Performed statistical analysis: K. Hashimoto. Wrote the main manuscript: K. Hashimoto. Gave suggestions on the manuscript: all authors.

## Supporting information

Figures S1–S3

Tables S1–S2

## Data Availability

The datasets generated and/or analyzed during the current study are available from the corresponding author on reasonable request, but no information infringing on the privacy of the participants will be given.
