# Supplementary figures and images for: Combined evaluation of ambulatory‐based late potentials and nonsustained ventricular tachycardia to predict arrhythmic events in patients with previous myocardial infarction: A Japanese noninvasive electrocardiographic risk stratification of sudden cardiac death (JANIES) substudy
Source: Ann Noninvasive Electrocardiol. 2020 Sep 24;26(1):e12803. doi: 10.1111/anec.12803 (PMC7816808; doi:10.1111/anec.12803)

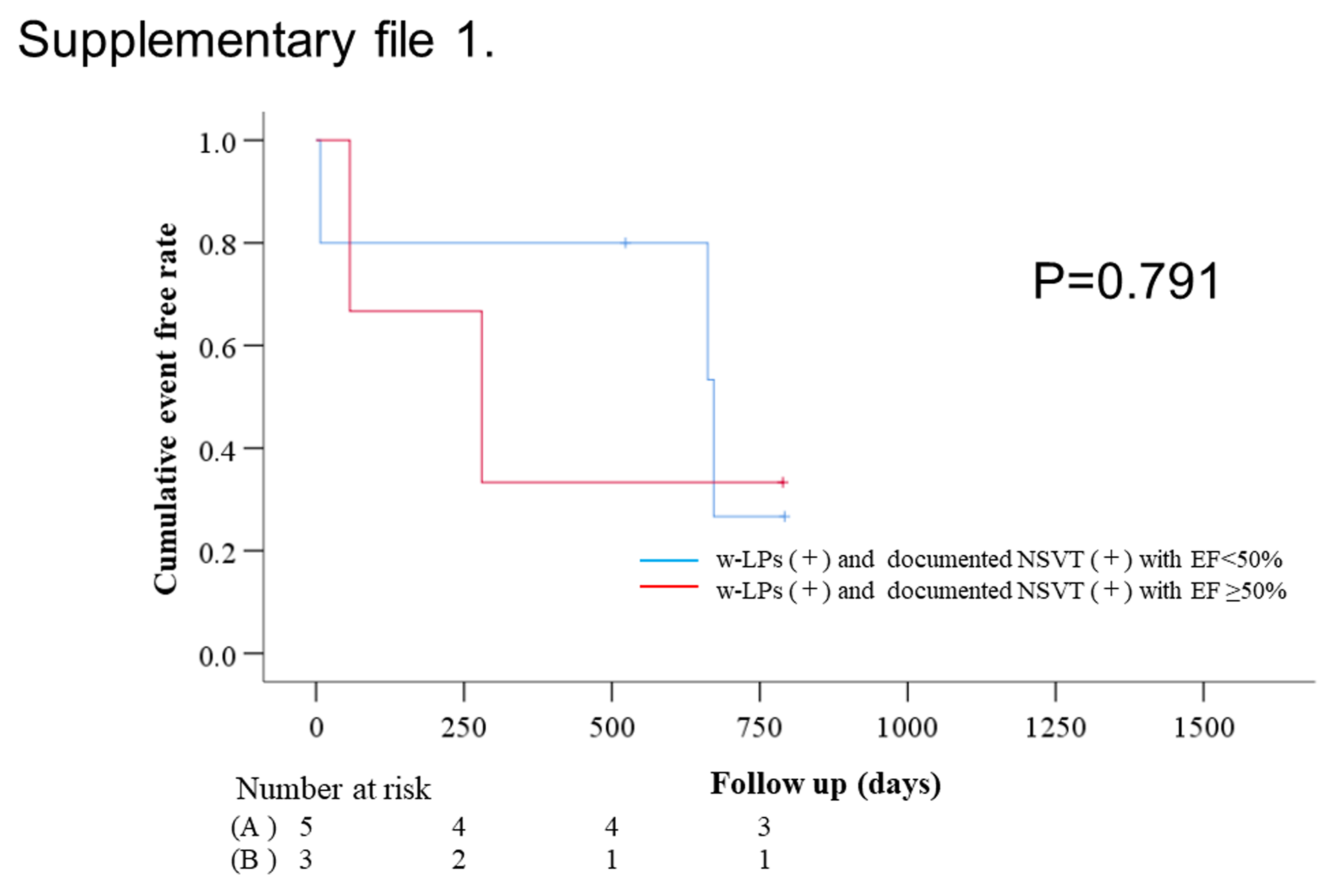


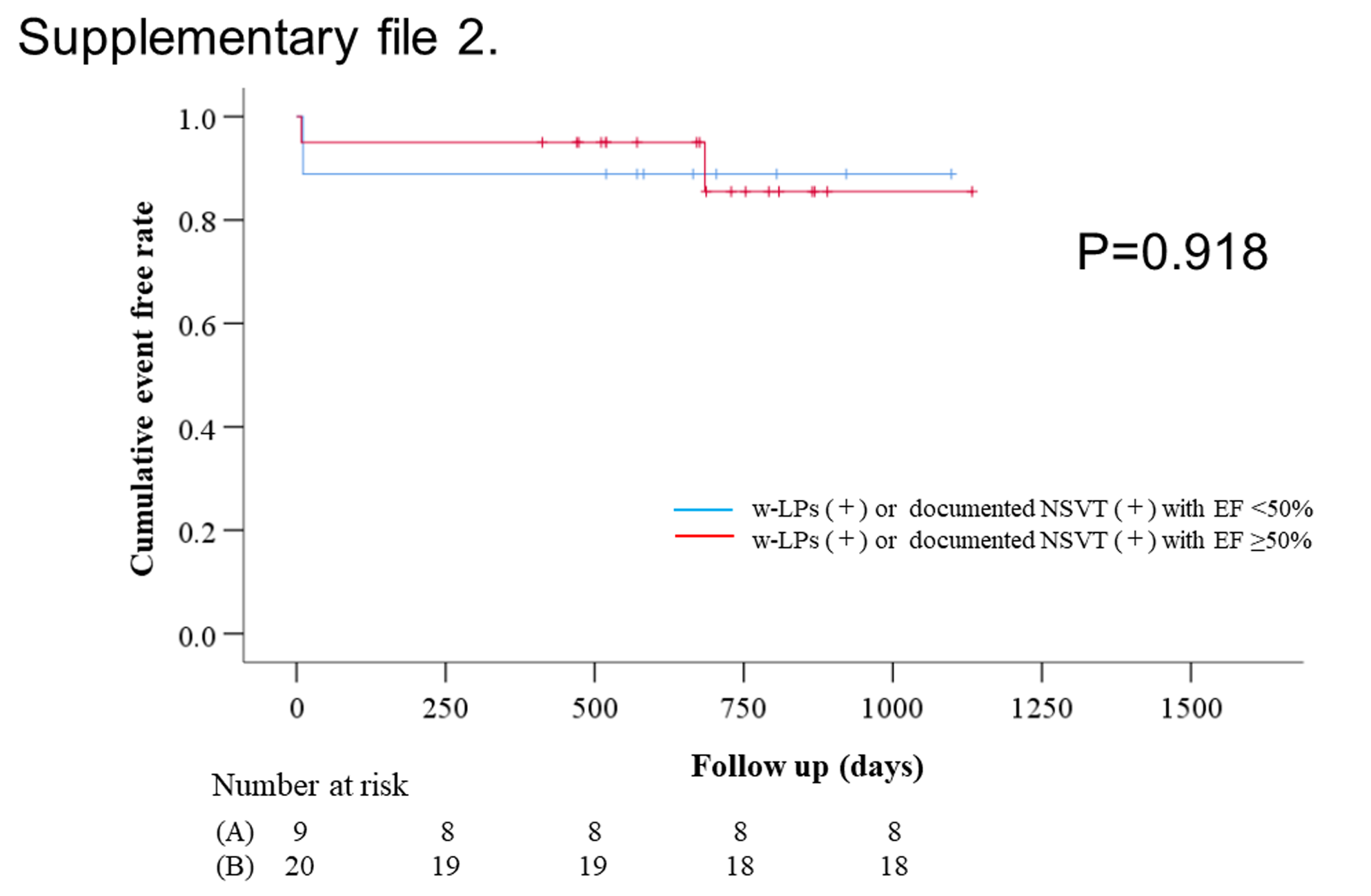


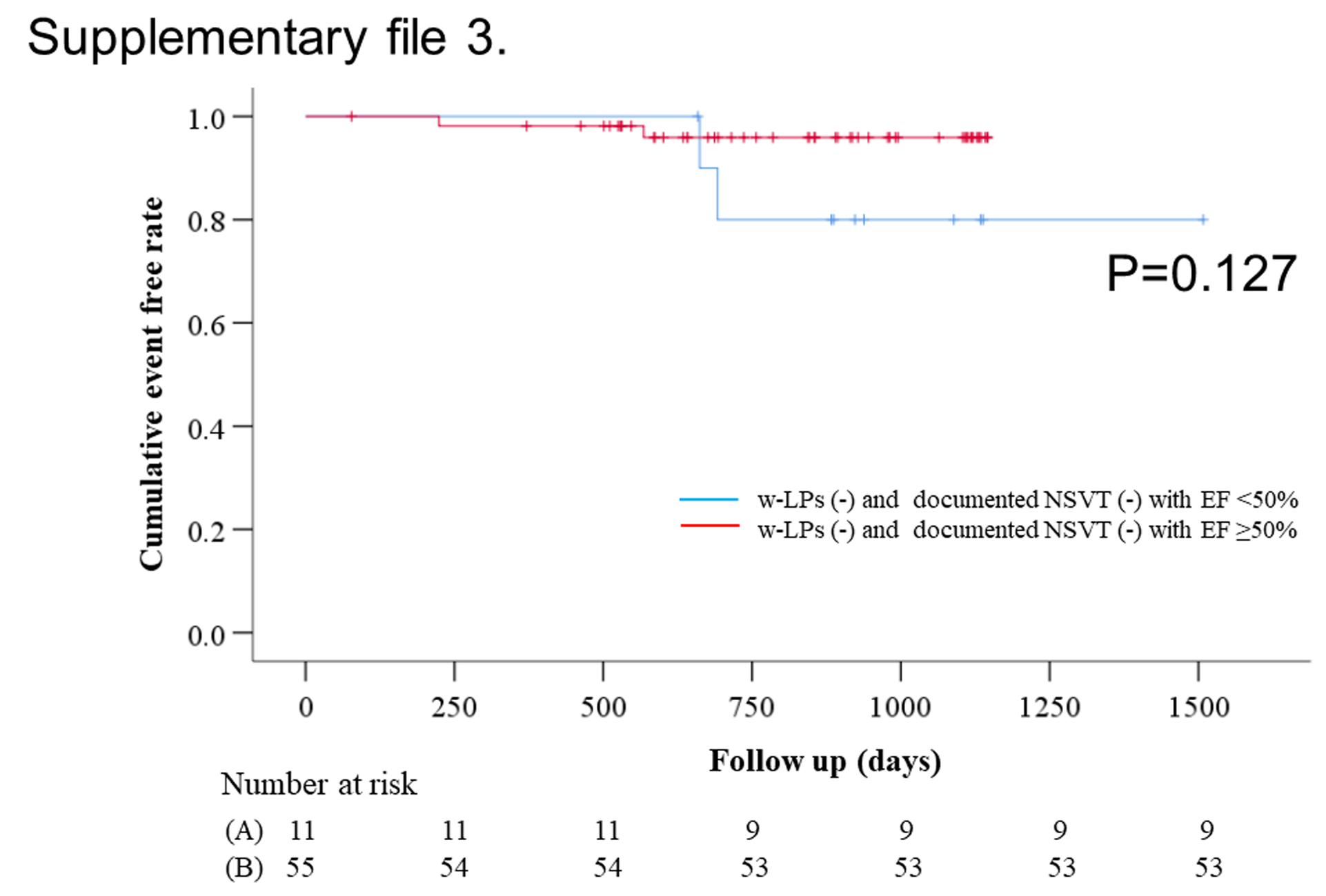

Supplement: Supplementary file 1 — Figures S1–S3 [file ANEC-26-e12803-s001.docx]
