# Supplementary material for: Combined evaluation of ambulatory‐based late potentials and nonsustained ventricular tachycardia to predict arrhythmic events in patients with previous myocardial infarction: A Japanese noninvasive electrocardiographic risk stratification of sudden cardiac death (JANIES) substudy
Source: Ann Noninvasive Electrocardiol. 2020 Sep 24;26(1):e12803. doi: 10.1111/anec.12803 (PMC7816808; doi:10.1111/anec.12803)
Supplement: Supplementary file 2 — Tables S1–S2 [file ANEC-26-e12803-s002.docx]

| Supplementary table 1. Result of multivariate Cox regression analysis (Model 1) | | |
| --- | --- | --- |
|  | Multivariate analysis | |
|  | Hazard ratio (95% CI) | P |
| b-LPs | 0.5 (0.1-2.5) | 0.38 |
| w-LPs | 1.0 (0.97-1.03) | 0.97 |
| HRT | 1.6 (0.4-6.1) | 0.46 |
| NSVT | 1.1 (0.1-9.4) | 0.96 |
| w-LPs＋NSVT | 17.6 (1.4-245.8) | 0.03 |
| NSVT=non-sustained ventricular tachycardia; w-LP=LP positive in worst value | | |
|  | | |

| Supplementary table 2. Result of multivariate Cox regression analysis (Model 2) | | |
| --- | --- | --- |
|  | Multivariate analysis | |
|  | Hazard ratio (95% CI) | P |
| w-LPs＋LVEF | 0.46 (0.03-6.4) | 0.56 |
| LVEF＋NSVT | 8.1 (1.0-62.3) | 0.045 |
| w-LPs＋NSVT | 12.2 (3.0-49.8) | <0.0001 |
| LVEF=left ventricular ejection fraction; | | |
| NSVT=non-sustained ventricular tachycardia; w-LPs=LP positive in worst value | | |
